# Review of Ceftazidime-Avibactam for the Treatment of Infections Caused by *Pseudomonas aeruginosa*

**DOI:** 10.3390/antibiotics10091126

**Published:** 2021-09-18

**Authors:** George L. Daikos, Clóvis Arns da Cunha, Gian Maria Rossolini, Gregory G. Stone, Nathalie Baillon-Plot, Margaret Tawadrous, Paurus Irani

**Affiliations:** 1 Department of Medicine, National and Kapodistrian University of Athens, 115-27 Athens, Greece; 2 Hospital Nossa Senhora das Graças, Curitiba 80810-040, PR, Brazil; arnscunha@gmail.com; 3 Department of Experimental and Clinical Medicine, University of Florence, I-50134 Florence, Italy; gianmaria.rossolini@unifi.it; 4 Clinical Microbiology and Virology Unit, Careggi University Hospital, I-50134 Florence, Italy; 5 Pfizer, Groton, CT 06340, USA; GregoryG.Stone@pfizer.com (G.G.S.); margaret.tawadrous@pfizer.com (M.T.); 6 Pfizer, CEDEX 14, 75688 Paris, France; nathalie.Baillon-Plot@pfizer.com; 7 Pfizer, Tadworth, Surrey KT20 7NS, UK; Paurus.Irani@pfizer.com

**Keywords:** ceftazidime–avibactam, *Pseudomonas aeruginosa*, multidrug resistance, complicated intra-abdominal infection, complicated urinary tract infection, hospital-acquired pneumonia

## Abstract

*Pseudomonas aeruginosa* is an opportunistic Gram-negative pathogen that causes a range of serious infections that are often challenging to treat, as this pathogen can express multiple resistance mechanisms, including multidrug-resistant (MDR) and extensively drug-resistant (XDR) phenotypes. Ceftazidime–avibactam is a combination antimicrobial agent comprising ceftazidime, a third-generation semisynthetic cephalosporin, and avibactam, a novel non-β-lactam β-lactamase inhibitor. This review explores the potential role of ceftazidime–avibactam for the treatment of *P. aeruginosa* infections. Ceftazidime–avibactam has good in vitro activity against *P. aeruginosa* relative to comparator β-lactam agents and fluoroquinolones, comparable to amikacin and ceftolozane–tazobactam. In Phase 3 clinical trials, ceftazidime–avibactam has generally demonstrated similar clinical and microbiological outcomes to comparators in patients with complicated intra-abdominal infections, complicated urinary tract infections or hospital-acquired/ventilator-associated pneumonia caused by *P. aeruginosa*. Although real-world data are limited, favourable outcomes with ceftazidime–avibactam treatment have been reported in some patients with MDR and XDR *P. aeruginosa* infections. Thus, ceftazidime–avibactam may have a potentially important role in the management of serious and complicated *P. aeruginosa* infections, including those caused by MDR and XDR strains.

## 1. Introduction

*Pseudomonas aeruginosa* is an opportunistic Gram-negative pathogen responsible for approximately 5–14% of all nosocomial or healthcare-associated infections and 16–40% of cases of ventilator-associated pneumonia (VAP) [1,2,3,4,5]. Patients with predisposing factors, such as severe burn victims, those with reduced immune function and those admitted to the intensive care unit (ICU), are at increased risk of acute *P. aeruginosa* infections; patients with cystic fibrosis (CF) or bronchiectasis may also develop chronic or recurrent *P. aeruginosa* infections [5,6,7,8,9]. While the reported extent of co-infection with bacterial pathogens in patients hospitalized with COVID-19 varies, *P. aeruginosa* is among the most frequently identified species in such patients, with a higher proportion in critically ill ICU patients [10]. Moreover, ventilated patients with COVID-19 may be at higher risk of developing VAP, with *P. aeruginosa* accounting for a high proportion of cases [11].

*P. aeruginosa* infections are often life-threatening and can be difficult to treat because these bacteria can express numerous acquired antimicrobial resistance mechanisms, virulence factors and mechanisms for evading host defences (including biofilm formation) [12,13,14]. Increasing antimicrobial resistance among *P. aeruginosa* (partly due to inappropriate antibiotic use) includes de novo development of resistance attributable to complex interactions between multiple adaptive cellular mechanisms and DNA mutations [15,16,17,18]. Healthcare practices continue to impact resistance profiles; it has been reported that global antimicrobial usage has surged during the COVID-19 pandemic, and there is concern about the impact this will have on resistance rates, particularly in ICUs, where *P. aeruginosa* is most problematic [19,20].

Antibiotics commonly used for *P. aeruginosa* infections include anti-pseudomonal cephalosporins, carbapenems, β-lactam/β-lactamase inhibitor combinations, fluoroquinolones, aminoglycosides and polymyxins [21]. Progressive accumulation of antibiotic resistance mechanisms may result in multidrug-resistant (MDR) and extensively drug-resistant (XDR) phenotypes of *P. aeruginosa* [8,12]. Although epidemiologically useful, the bedside applicability of MDR and XDR definitions is limited, as their definitions require resistance to only one agent per antimicrobial category, and all antibiotics are weighted equally regardless of effectiveness and toxicity [22]. Consequently, difficult-to-treat resistance (DTR), defined as in vitro resistance to all first-line agents, has been proposed to describe antimicrobial resistance in Gram-negative bacteria [22]. *P. aeruginosa* was the most frequently identified DTR pathogen (accounting for 38.1% of 1371 episodes) in a recent retrospective review of Gram-negative infections in US hospitals [23]. The development of MDR and DTR *P. aeruginosa* is of particular concern for public health and highlights the need for novel therapeutics to treat *P. aeruginosa* infections [8,12,24].

Ceftazidime–avibactam is a combination antimicrobial agent comprising ceftazidime (an extended-spectrum, third-generation, semisynthetic anti-pseudomonal cephalosporin) and avibactam (a novel non-β-lactam β-lactamase inhibitor) [25,26,27,28]. Ceftazidime has broad in vitro activity against Gram-negative aerobic bacteria, including *P. aeruginosa* and *Enterobacterales* [29]. However, the utility of third-generation cephalosporins has become compromised by the increasing prevalence of MDR Gram-negative bacteria, including those producing extended-spectrum β-lactamases (ESBLs), chromosomal AmpC cephalosporinases, *Klebsiella pneumoniae* carbapenemases (KPCs) and metallo-β-lactamases (MBLs) [30]. Avibactam restores the in vitro activity of ceftazidime against bacteria expressing Ambler class A (e.g., ESBLs and KPCs), class C (e.g., AmpC cephalosporinases), including some that co-express class D (e.g., oxacillinase-48) β-lactamases, but not against those that produce MBLs [25,26,30]. Ceftazidime–avibactam is active in vitro against many MDR, ceftazidime-non-susceptible and carbapenem-resistant *P. aeruginosa* strains [31,32,33,34].

This mini-review explores the potential role of ceftazidime–avibactam in the management of *P. aeruginosa* infections.

## 2. Antimicrobial Resistance in *P. aeruginosa*

*P. aeruginosa*, a ubiquitous organism with a relatively large genome and flexible metabolic capabilities, can exploit numerous environmental niches [35,36]. *P. aeruginosa* displays a formidable array of resistance mechanisms including efflux pumps, porin mutations and enzymes that confer resistance to β-lactam and aminoglycoside antibiotics [37,38,39,40]. Its intrinsic resistance to many antibiotics is largely due to the low permeability of its outer membrane, which limits drug penetration [41], and at least five families of efflux pumps that actively extrude antibiotics (e.g., MexA-MexB-OprM) have been identified in *P. aeruginosa* [42,43]. In addition, *P. aeruginosa* can acquire multiple resistance mechanisms through chromosomal gene mutations and horizontal transfer of mobile genetic elements, including plasmids, transposons, integrons, prophages and resistance islands, via conjugation, transformation or transduction [41,43]. In *P. aeruginosa*, horizontal gene transfer primarily affects aminoglycoside and β-lactam resistance but has been reported for other antibiotic classes, including fluoroquinolones [41,44,45,46,47]. *P. aeruginosa* biofilms are associated with persistent infections that are often recalcitrant to host defences and antibiotic therapy [35,48].

Although anti-pseudomonal β-lactams such as ceftazidime play an important part in treatment of *P. aeruginosa* infections, acquired β-lactamases in *P. aeruginosa* including ESBLs and carbapenemases can hydrolyse many β-lactams, including broad-spectrum cephalosporins and monobactams [49]. *P. aeruginosa* carries a chromosomal drug-inducible gene, *ampC*, which encodes AmpC, a broad-spectrum class C β-lactamase [50]. Wild-type *P. aeruginosa* typically express AmpC constitutively at low levels [38]. However, in the presence of an inducing β-lactam, AmpC expression may increase 100- to 1000-fold, greatly increasing resistance to antipseudomonal penicillins (ticarcillin and piperacillin), monobactams (aztreonam) and third- (ceftazidime) and fourth-generation (cefepime) cephalosporins [50,51]. Regulation of *ampC* expression is controlled by three major gene products, namely an inner membrane permease (AmpG); a cytosolic amidase (AmpD); and a positive transcriptional regulator (AmpR), belonging to the LysR family [38]. Overproduction of AmpC is associated with mutations arising in *ampG*, *ampD* and *ampR* [52,53]. Some *P. aeruginosa* isolates exhibit hyperproduction of the chromosomal β-lactamase caused by mutations in the regulatory circuit that controls the β-lactamase-inducible gene, *ampC*, and thus greatly increases the resistance to cephalosporins such as ceftazidime [50]. *P. aeruginosa* can also develop resistance to carbapenems through mutations down-regulating the expression of membrane porins, by upregulation of some efflux systems (e.g., MexAB-OprM) and through the acquisition of transferable genes encoding carbapenemases, such as MBLs (mostly VIM, IMP and occasionally NDM), KPCs and Guiana extended-spectrum (GES) enzymes [54,55].

For 2019, data from the European Antimicrobial Resistance Surveillance Network showed that 31.8% of >20,000 *P. aeruginosa* isolates from 30 European countries were resistant to at least one of five antimicrobial groups (piperacillin ± tazobactam, fluoroquinolones, ceftazidime, aminoglycosides and carbapenems). Resistance to two or more antimicrobial groups was found in 17.6% of isolates, and 3.4% were resistant to all five antimicrobial groups [56]. These data showed encouraging population-weighted mean trends of declining overall *P. aeruginosa* resistance across Europe compared with previous years [56,57]; however, resistance rates vary substantially among countries, with high rates prevalent in eastern and southern countries.

In the US, the antimicrobial susceptibility of 7452 *P. aeruginosa* isolates collected from 79 medical centres in 2012–2015 was evaluated as part of the International Network for Optimal Resistance Monitoring (INFORM) programme [33]. MDR and XDR *P. aeruginosa* phenotypes were observed among 15.4% and 9.4% of isolates, respectively [33]. In China, the percentage of *P. aeruginosa* strains isolated from patients hospitalized in burn wards increased annually from 10.2% in 2007 to 26.2% in 2014, with this species becoming the predominant one among Gram-negative bacteria by 2014 [58]. Over the study period, the proportion of MDR *P. aeruginosa* increased from 64.0% in 2007 to 89.9% in 2014 [58].

## 3. Treatment Guidelines for Management of *P. aeruginosa* Infections

### 3.1. Antibiotics for P. aeruginosa Infections

Antimicrobial agents commonly used for the treatment of *P. aeruginosa* infections include intravenous (IV) β-lactams (such as anti-pseudomonal cephalosporins, carbapenems and β-lactam/β-lactamase inhibitor combinations), fluoroquinolones and aminoglycosides, as well as polymyxins (colistin) in cases of last resort. Depending on the site and severity of infection, as well as the local resistance epidemiology, treatment guidelines for empiric and definitive antibiotic therapy of suspected or confirmed *P. aeruginosa* infections recommend various monotherapy or combination regimens using agents from the above classes (Table 1). Surgical source control is also recommended for patients with intra-abdominal infection (IAI). Some of the more recently published guidelines include guidance for use of ceftolozane–tazobactam and/or ceftazidime–avibactam for certain *P. aeruginosa* infections [59,60,61,62,63].

**Table 1:** Antimicrobial therapy recommendations for common *P. aeruginosa* infections.

Guideline	Clinical Indication(s)	Antimicrobial Agent(s)	Recommendation(s)
European Association of Urology (EAU), 2018 [59]	cUTI, including pyelonephritis and urosepsis	Ceftazidime Cefepime Piperacillin/tazobactam Ceftolozane/tazobactam Ceftazidime/avibactam Gentamicin * Amikacin * Imipenem/cilastatin Meropenem	Treatment options for empirical antimicrobial therapy. The choice between these agents should be based on local resistance data, and the regimen should be tailored on the basis of susceptibility results. Amoxicillin, co-amoxiclav, trimethoprim and trimethoprim–sulphamethoxazole and fluoroquinolones should not be used as empiric treatment for urological patients.
British Society for Antimicrobial Chemotherapy (BSAC)/Healthcare Infection Society (HIS)/British Infection Association (BIA), 2018 [60]	UTI, IAI	Ceftazidime Piperacillin/tazobactam Carbapenems (excluding ertapenem) Aminoglycosides Fluoroquinolones Ceftolozane/tazobactam	Personalize empirical chemotherapy for each patient by considering current features of bacteraemia, risk factors for antibiotic resistance and past susceptibility testing, including the presence of MDR GNB in the patient, hospital unit, nursing home or community. Do not use imipenem to treat susceptible *Pseudomonas* infections. Do not use ceftolozane/tazobactam for infections due to AmpC- or CPE or MBL/ESBL-producing *P. aeruginosa.*
World Society for Emergency Surgery (WSES), 2017 [64]	cIAI	Piperacillin/tazobactam Imipenem/cilastatin Doripenem Ciprofloxacin/levofloxacin1 Ceftazidime Cefepime Ceftazidime–avibactam Ceftolozane–tazobactam Amikacin Gentamicin Colistin	In critically ill patients, antimicrobial therapy should be started as soon as possible. In these patients, to ensure timely and effective administration of antibiotics, clinicians should always consider the pathophysiological status of the patient as well as the PK properties of the employed antibiotics.
Surgical Infection Society (SIS), 2017 [65]	cIAI	Ceftolozane–tazobactam Aminoglycosides Polymyxin	Empirical treatment options for patients with risk factors for MDR, XDR or PDR *P. aeruginosa* (± coverage for *Staphylococcus aureus*).
Spanish Society of Chemotherapy, 2018 [61]	Acute invasive infections	Ceftolozane–tazobactam Ceftazidime/avibactam Meropenem Ceftazidime Piperacillin/tazobactam + Amikacin, colistin or ciprofloxacin	Include a β-lactam with activity against *P. aeruginosa* with (a) the highest probability to achieve the optimal value of the adequate pharmacokinetic/pharmacodynamic index, and (b) the lowest risk of selection/amplification of the resistant subpopulation. For empirical treatment, consider combination antibiotics during the first 48–72 h to rapidly decrease the bacterial population, avoid selection of resistance and increase the probability of the strain to be susceptible at least to one of the two antibiotics. For directed treatment schedules, consider combination antibiotics if the infection presents criteria for severe sepsis or septic shock, in central nervous system infections, in endocarditis or neutropenia and when *P. aeruginosa* is resistant to β-lactams. Whatever antibiotic is chosen, it is essential to optimize the dose and route of administration. Preferred treatment for patients with severe sepsis/septic shock * and/or with risk factors for MDR *P. aeruginosa* infections.
Infectious Diseases Society of America (IDSA), 2020 [63]	cUTI including pyelonephritis	Ceftolozane–tazobactam Ceftazidime–avibactam Imipenem–cilastatin–relebactam Cefiderocol	Preferred treatment options for pyelonephritis and cUTI caused by DTR- *P. aeruginosa*.
	DTR *P. aeruginosa* infections outside the urinary tract	Ceftolozane–tazobactam Ceftazidime–avibactam Imipenem–cilastatin–relebactam	Preferred treatment options (as monotherapy) for the treatment of infections outside of the urinary tract caused by DTR- *P. aeruginosa.*
American Thoracic Society (ATS)/Infectious Diseases Society of America (IDSA), 2016 [66]	HAP/VAP	Piperacillin–tazobactam Cefepime Ceftazidime Imipenem Meropenem Aztreonam Fluoroquinolones Aminoglycosides Colistin	Empiric regimens should cover for *S. aureus*, *P. aeruginosa* and other Gram-negative bacilli. For patients with VAP or HAP with high mortality risk, include two anti-pseudomonal antibiotics from different classes. In units where >10% of Gram-negative isolates are resistant to an agent being considered for monotherapy, and patients in ICUs where local antimicrobial susceptibility rates are unavailable ^†^. Treat with two anti-pseudomonal agents of different classes for patients with risk factors for *P. aeruginosa* or other Gram-negative infection or is at high risk of mortality ^‡^.
European Respiratory Society (ERS)/European Society of Intensive Care Medicine (ESICM)/European Society of Clinical Microbiology and Infectious Diseases (ESCMID)/Latin American Thoracic Association (ALAT), 2017 [67]	HAP/VAP	Cefepime Ceftazidime Piperacillin/tazobactam Imipenem Meropenem Levofloxacin	Consider a risk-stratification based approach; all empiric therapy regimens for HAP/VAP should include anti- pseudomonal coverage. Dual anti-pseudomonal antimicrobial empiric therapy (± coverage for *S. aureus*) recommended for patients with septic shock and in settings with high MDR pathogen risk.
UK National Institute for Health and Care Excellence (NICE), 2019 [62]	HAP/VAP	Piperacillin/tazobactam Anti-pseudomonal cephalosporins Meropenem Ceftazidime–avibactam	Treat with broad-spectrum empiric Gram-negative coverage (±coverage for *S. aureus*).
European Cystic Fibrosis Society (ECFS), 2018 [68]	CF	Tobramycin solution (or dry powder) for inhalation Aztreonam inhalation solution Combination of nebulized colistin and oral ciprofloxacin	Treatment options for new and chronic bronchopulmonary *P. aeruginosa* infections.

* Severity criteria include criteria of severe sepsis or septic shock, severe immunodepression (especially neutropenia < 500 cells/mm^3^) and infections involving high bacterial load, being not surgically controllable, such as extensive pneumonia or pneumonia with cavitations/necrosis. ^†^ For empirical antimicrobial therapy for patients with clinically suspected VAP. ^‡^ For empirical antimicrobial therapy for patients with clinically suspected HAP. CF, cystic fibrosis; cIAI, complicated intra-abdominal infection; cUTI, complicated urinary tract infection; DTR, difficult-to-treat resistance; HAP, hospital-acquired pneumonia; IAI, intra-abdominal infection; MDR, multidrug-resistant; PDR, pandrug-resistant; UTI, urinary tract infection; VAP, ventilator-associated pneumonia; XDR, extensively drug-resistant.

### 3.2. Complicated Intra-Abdominal Infections 

The Surgical Infection Society guidelines for management of IAIs (2017) include stratification of empiric antimicrobial treatment recommendations based on the risk of pseudomonal involvement [65]. Third- or fourth-generation cephalosporins (e.g., cefotaxime, ceftriaxone, ceftizoxime, ceftazidime and cefepime) in combination with metronidazole, β-lactam/β-lactamase inhibitors (e.g., piperacillin/tazobactam and ticarcillin/clavulanic acid) and carbapenems (e.g., meropenem and imipenem/cilastatin) are commonly used [69]. For patients with complicated IAI (cIAI) considered at risk for infection with MDR, XDR or pandrug-resistant *P. aeruginosa*, combinations of a β-lactam antibiotic, including ceftolozane–tazobactam, an aminoglycoside and/or a polymyxin are recommended [65]. The World Society for Emergency Surgery guidelines (2017) provide similar recommendations and, also recognize ceftolozane–tazobactam and ceftazidime–avibactam as approved treatments for cIAI caused by *P. aeruginosa* [64].

### 3.3. Complicated Urinary Tract Infections 

The European Association of Urology (EAU) and the Dutch Working Party on Antibiotic Policy recommend antimicrobial treatment options for patients with complicated urinary tract infection (cUTI) that provide coverage against *P. aeruginosa* and include amoxicillin plus an aminoglycoside, a second-generation cephalosporin plus an aminoglycoside or a third-generation cephalosporin [59,70]. A carbapenem with anti-pseudomonal activity (imipenem/meropenem) was recommended in 2013 for empiric therapy in patients with risk factors for ESBL infections [70]. The most recent EAU guidelines (2018) include recommendations for ceftazidime–avibactam and ceftolozane–tazobactam as empiric treatment options for pyelonephritis (second line) and urosepsis [59].

### 3.4. Hospital-Acquired Pneumonia and Ventilator-Associated Pneumonia

In patients with suspected VAP, the American Thoracic Society (ATS)/Infectious Diseases Society of America (IDSA) guidelines (2016) recommend coverage for *Staphylococcus aureus*, *P. aeruginosa* and other Gram-negative bacilli in all empiric regimens [66]. Combination therapy with two anti-pseudomonal antibiotics from two different classes is recommended in units where >10% of Gram-negative isolates are resistant to an agent being considered for monotherapy, and patients in ICUs where local antimicrobial susceptibility rates are not available. For patients with hospital-acquired pneumonia (HAP) who are being treated empirically, ATS/IDSA guidelines recommend antibiotics with coverage against *P. aeruginosa* and other Gram-negative bacilli. If a patient has risk factors that increase the likelihood of *P. aeruginosa* or other Gram-negative infection (e.g., prior antimicrobials within 90 days), or is at high risk of mortality (e.g., need for ventilatory support due to HAP and septic shock), combination therapy with two anti-pseudomonal agents of different classes is recommended [66]. 

Similarly, international guidelines for management of patients with HAP/VAP (2017) published by the European Respiratory Society, European Society of Clinical Microbiology and Infectious Diseases, European Society of Intensive Care Medicine and Latin American Thoracic Association recommend a risk-stratification based approach, with dual anti-pseudomonal antimicrobial empiric therapy (±coverage for *S. aureus*) for high-risk patients including those with septic shock and in settings with high MDR pathogen risk [67]. For patients with HAP/VAP and severe signs or symptoms, or those at higher risk of resistance, the UK National Institute for Health and Care Excellence guidelines (2019) do not specify dual-antipseudomonal treatment but include broad-spectrum empiric Gram-negative coverage (±coverage for *S. aureus*) with recommended agents including piperacillin/tazobactam, antipseudomonal cephalosporins, meropenem and ceftazidime–avibactam [62].

### 3.5. Cystic Fibrosis 

The impaired mucociliary clearance in patients with CF provides a microenvironment in which pathogenic bacteria, including *P. aeruginosa*, can become the source of chronic pulmonary infections. The incidence of chronic *P. aeruginosa* infection in people with CF patients increases with age, and such individuals have worse health status and experience more rapid disease progression than age-matched controls [71]. The European Cystic Fibrosis Society recommendations for the management of new and chronic *P. aeruginosa* infections in patients with CF (2018) include tobramycin solution or dry powder for inhalation, aztreonam inhalation solution or a combination of nebulized colistimethate and oral ciprofloxacin; the aim of treatment should be eradication (documented by follow-up cultures) [68]. Acute exacerbations of pulmonary infections in patients with CF can result in hospitalization and require IV antibiotic treatment [68].

## 4. Role of Ceftazidime–Avibactam in the Treatment of *P. aeruginosa* Infections

### 4.1. Approved Indications

In Europe and the US, ceftazidime–avibactam is approved for the treatment of adults cUTI (including pyelonephritis), cIAI (in combination with metronidazole) and HAP (including VAP), including bacteraemia associated with these infections caused by susceptible *P. aeruginosa* and *Enterobacterales*. In Europe, it is also approved for infections caused by aerobic Gram-negative organisms in adult patients with limited treatment options. European and US approvals were recently extended to include paediatric patients ≥3 months old (cUTI and cIAI indications only in the US) [72,73].

### 4.2. Mechanism of Action

β-lactams, including ceftazidime, exert antimicrobial effects through binding to penicillin binding proteins in bacterial cell walls, thereby disrupting cell wall synthesis and bacterial growth. As noted above, numerous antimicrobial resistance mechanisms can be expressed by MDR *P. aeruginosa*. Anti-pseudomonal cephalosporins such as ceftazidime, cefepime and ceftolozane have lower affinity for AmpC cephalosporinases (commonly expressed by *P. aeruginosa*) and additional stability against enzymatic hydrolysis than other cephalosporins [74,75]. However, susceptibility to these agents can be reduced by hyperexpression of AmpC. The addition of avibactam to ceftazidime overcomes AmpC cephalosporinase-mediated ceftazidime resistance among *P. aeruginosa* isolates in vitro (including those co-expressing EBSLs), but the combination is unable to overcome resistance mediated by porin mutations, efflux pumps or MBLs [76,77,78,79]. Moreover, alterations in AmpC-encoding and control genes conferring reduced susceptibility to ceftazidime–avibactam, ceftolozane/tazobactam and carbapenems have been reported in laboratory studies and/or identified in *P. aeruginosa* clinical isolates from patients undergoing antimicrobial therapy [50,80,81,82,83]. These findings are a salient reminder of the propensity of *P. aeruginosa* to undergo rapid evolution to develop novel resistance phenotypes and highlight the vital importance of microbiological cultures, susceptibility testing (as well as local and regional susceptibility patterns) and use of molecular diagnostics wherever possible to guide treatment of *P. aeruginosa* infections. In vitro data suggest that combining ceftazidime–avibactam with other antibiotics such as aminoglycosides or colistin may be synergistic against MDR *P. aeruginosa* [84,85,86]. 

### 4.3. In Vitro Activity

Numerous international and regional antimicrobial surveillance studies have reported on the in vitro activity of ceftazidime–avibactam using Clinical and Laboratory Standards Institute (CLSI) and European Committee on Antimicrobial Susceptibility Testing (EUCAST) interpretative criteria (also referred to as minimum inhibitory concentration (MIC) breakpoints), which define isolates of *P. aeruginosa* and *Enterobacterales* with ceftazidime–avibactam MICs ≤8 mg/L as susceptible [87,88]. Key susceptibility data for *P. aeruginosa* are summarized in Table 2.

**Table 2:** Overview of key in vitro studies of ceftazidime–avibactam activity against clinical isolates of *P. aeruginosa*.

Study	Number of Isolates, Region and Study Dates	Isolate Source(s)	Agent	MIC Range, mg/L	MIC_50_, mg/L	MIC_90_, mg/L	Percentage Susceptible, %	Ceftazidime–Avibactam Resistance Mechanisms
Nichols et al. (2016) [89]	7062 Asia/South Pacific, Europe, Latin America, Middle East/Africa (2012–2014)	NR	Ceftazidime–avibactam	≤0.5 to >128	4	8	92.0	MBLs (VIM, IMP, NDM), serine carbapenemases (KPC-2, GES) and ESBLs (SHV-5, VEB, PER, GES ESBL-like, TEM-OSBL)
			Ceftazidime	≤0.5 to >128	2	64	77.0	
Kazmierczak et al. (2016) [90]	8010 Asia Pacific, Europe, Latin/North America, Middle East/Africa 2012–2014)	Intra-abdominal, urinary tract, skin and soft tissue, lower respiratory tract and bloodstream infections	Ceftazidime–avibactam	0.06 to >128	2	8	92.4	KPC-2, VIM-2, AmpC
			Ceftazidime	0.06 to >128	2	64	77.4	
Kazmierczak et al. (2016) [90]	29 (KPC- positive) Asia Pacific, Latin America (2012–2014)	Intra-abdominal, urinary tract, skin and soft tissue, lower respiratory tract and bloodstream infections	Ceftazidime–avibactam	4 to 64	8	32	75.9	KPC-2, VIM-2, AmpC
			Ceftazidime	64 to 128	64	>128	0.0	
Sader et al. (2017) [79]	7868 North America (2013–2016)	Intrabdominal, urinary tract, skin and skin structure, pneumonia, bloodstream and other infection types	Ceftazidime–avibactam	0.25 to >32	2	4	97.1	NR
			Ceftazidime	NR	2	32	84.7	
Sader et al. (2017) [79]	1562 (MDR) North America (2013–2016)	Intra-abdominal, urinary tract, skin and skin structure, pneumonia, bloodstream and other infection types	Ceftazidime–avibactam	0.25 to >32	4	16	86.5	NR
			Ceftazidime	NR	16	>32	43.6	
Sader et al. (2017) [91]	3402 North America (2011–2015)	Pneumonia	Ceftazidime–avibactam	0.25 to >32	2	4	96.6	NR
			Ceftazidime	NR	2	32	82.4	
Atkin 2018 [92]	32 North America (2015)	Cystic fibrosis	Ceftazidime–avibactam	0.5 to >128	4	64	71.9	OprD protein loss, AmpC, MexC MexX MexA
			Ceftazidime	16 to >128	64	>128	0.0	
Sader et al. (2019) [93]	2215 North America (2017–2018)	Pneumonia	Ceftazidime–avibactam	≤0.015 to >32	2	8	96.0	NR
			Ceftazidime	NR	2	32	79.8	
Sader et al. (2019) [93]	526 (MDR) North America (2017–2018)	Pneumonia	Ceftazidime–avibactam	0.06 to >32	4	16	83.5	NR
			Ceftazidime	NR	32	>32	32.3	
Sid Ahmed et al. (2019) [94]	205 (MDR) Middle East (2014–2016)	Respiratory tract, skin and soft tissue, urinary tract, bloodstream, sterile body fluids and vascular line tips	Ceftazidime–avibactam	≤0.75 to >256	4	64	68.8	MBLs (VIM-2 type) and ESBLs (VEB-1a, OXA-4, OXA-10, OXA-50, TEM-116, PDC-2, PDC-3, PDC-5 and PDC-7)

ESBL, extended-spectrum β-lactamase; GES, Guiana extended-spectrum; IMP, imipenemase; KPC, *Klebsiella pneumoniae* carbapenemase; MIC, minimum inhibitory concentration; MBL, metallo-β-lactamase; MDR, multidrug-resistant; MexA, Mex C, MexX, Mex-family efflux pumps; NDM, New Delhi metallo-β-lactamase; NR, not reported; OSBL, original spectrum β-lactamase; OXA, oxacillinase; PDC, Pseudomonas-derived cephalosporinase; SHV, sulfhydryl variable; TEM, Timoniera; VEB, Vietnamese extended-spectrum β-lactamase; VIM, Verona integron-encoded metallo-β-lactamase.

Antimicrobial susceptibility testing was performed for ceftazidime–avibactam and comparator agents against 7062 clinical isolates of *P. aeruginosa* collected during 2012–2014 in four geographic regions (Europe, Asia/South Pacific, Latin America and Middle East/Africa) as part of the INFORM global surveillance study. The majority of isolates were susceptible (88.7–93.2%) to ceftazidime–avibactam across the four regions (MIC_90_ values of 8–16 mg/L), in contrast to lower susceptibilities among comparator β-lactams: ceftazidime (MIC_90_, 32–64 mg/L; 71.5–80.8% susceptible), meropenem (MIC_90_, >8 mg/L; 64.9–77.4% susceptible) and piperacillin–tazobactam (MIC_90_, >128 mg/L; 62.3–71.3% susceptible) [89].

In an analysis of 11,185 Gram-negative isolates from hospitalized patients with pneumonia (including VAP) in 76 US medical centres between 2011 and 2015, ceftazidime–avibactam displayed good in vitro activity against 3402 *P. aeruginosa* isolates (MIC_50_/MIC_90_, 2 and 4 mg/L; 96.6% susceptible), including isolates non-susceptible to meropenem (86.3% susceptible to ceftazidime–avibactam), piperacillin–tazobactam (85.6% susceptible) or ceftazidime (80.6% susceptible). Other agents that were active against *P. aeruginosa* included colistin (MIC_50_/MIC_90_, 1 and 2 mg/L; 99.6% susceptible) and amikacin (MIC_50_/MIC_90_, 4 and 16 mg/L; 95.3% susceptible) [91].

Bacterial pathogens expressing KPC are clinically significant, as they frequently co-express multiple other resistance mechanisms [90]. The gene encoding KPC (*bla*_KPC_) has been observed in multiple *Enterobacterales* species and some non-fermentative Gram-negative pathogens, including *P. aeruginosa* [90]. Among 8010 *P. aeruginosa* isolates collected in 40 countries as part of the INFORM global surveillance study (2012–2014), 29 carbapenem-non-susceptible isolates carried *bla*_KPC_ (*K. pneumoniae* was the most commonly isolated KPC-producing species). The majority of antimicrobial agents tested were inactive against 29 KPC-positive *P. aeruginosa* isolates (susceptibilities of <4%). However, 75.9% of these isolates were susceptible to ceftazidime–avibactam (MIC_90_, 32 mg/L). Other agents that were active against KPC-positive *P. aeruginosa* included colistin (MIC_90_, 2 mg/L; 96.6% susceptible) and amikacin (MIC_90_, >32 mg/L; 75.9% susceptible) [90].

Similarly, against 7868 *P. aeruginosa* isolates from 94 US hospitals (2013–2016), ceftazidime–avibactam showed good in vitro activity (MIC_90_, 4 mg/L; 97.1% susceptible), including against MDR isolates (MIC_90_, 16 mg/L; 86.5% susceptible) and inhibited (MIC <8 mg/L) 71.8% of 628 isolates that were non-susceptible to meropenem, piperacillin–tazobactam and ceftazidime [79].

Resistance mechanisms identified among *P. aeruginosa* isolates in the studies in Table 2 were predominantly enzymatic and included MBLs, serine carbapenemases (e.g., KPC-2 and GES) and ESBLs (e.g., SHV-5, VEB, PER and GES; Table 2); of note, non-enzymatic mechanisms, such as overexpression of efflux pumps and porin mutations, were not assessed in some studies [89,90,94]. In the 2012–2014 analysis, 563 of 7062 (8%) *P. aeruginosa* isolates were resistant to ceftazidime–avibactam, of which 291 (51.7%) were MBL-positive, 21 carried genes for serine carbapenemases (KPC-2 or GES type) ±ESBLs, one isolate carried a GES of undefined activity, and 51 isolates harboured only ESBLs (SHV-5, VEB type, PER type or GES type); no acquired β-lactamase gene was identified in the remaining 199 ceftazidime–avibactam-resistant isolates [89]. Thus, approximately 271 non-MBL-expressing isolates expressed other unidentified resistance mechanisms that were not detected by PCR amplification and gene sequencing [89].

Analogous to ceftazidime–avibactam, ceftolozane-tazobactam, a β-lactam–β-lactamase inhibitor with in vitro activity against *P. aeruginosa* (including MDR isolates), is approved in Europe and the US for the treatment of adults with cUTI, cIAI and HAP/VAP [95,96]. Both ceftazidime–avibactam and ceftolozane–tazobactam have similar good activity against *P. aeruginosa* [93,94,97]. Other recently developed agents with activity against MDR *P. aeruginosa* (including some isolates resistant to ceftazidime–avibactam and ceftolozane–tazobactam) include cefiderocol, imipenem/cilastatin–relebactam and cefepime–zidebactam [98,99,100].

### 4.4. Pharmacokinetics and Pharmacodynamics

Population pharmacokinetic (PK) and pharmacodynamic (PD) analyses and probability of target attainment (PTA) simulations using an iterative modelling approach encompassing additional data at various points during clinical development supported the selection of doses for Phase 2 and 3 evaluation (including adjustments for renal impairment) and the determination of MIC susceptibility breakpoints for target pathogens, including *P. aeruginosa* [101,102,103,104].

For β-lactam antibiotics, the primary driver of PD is the amount of time free drug concentrations are maintained above the MIC of the target pathogen (%*f*T > MIC). For ceftazidime, the β-lactam component of ceftazidime–avibactam, 50% *f*T > MIC is the established PK/PD target based on neutropenic mouse infection models and is associated with microbiological eradication in patients with Gram-negative infections [101]. In global surveillance studies, ceftazidime–avibactam MIC_90_ values of ≤8 mg/L were reported for phenotypically and genotypically unselected clinical isolates of *P. aeruginosa* [101]. Therefore, a target plasma concentration of 8 mg/L (i.e., matching the upper MIC_90_ value for target pathogens from contemporary surveillance studies) was selected for the ceftazidime component of the joint PK/PD target [101].

Based on in vitro hollow fibre and in vivo data, a PK/PD index for avibactam in combination with ceftazidime defined as percentage of time that free drug concentrations exceed a threshold concentration (C_T_) of 1 mg/L over a dose interval (%*f*T > CT) (in combination with ceftazidime) was associated with bacteriostasis in a *P. aeruginosa* neutropenic mouse thigh infection model and 2-log_10_ killing in a *P. aeruginosa* neutropenic mouse lung infection model [101,105,106]. Accordingly, joint attainment of 50% *f*T > 8 mg/L for ceftazidime and 50% *f*T > 1 mg/L for avibactam, to be achieved simultaneously, was considered as the main PK/PD target for the PTA analyses.

The final population PK models, which included ceftazidime and avibactam PK data from eighteen Phase 1–3 clinical trials, were used to predict steady-state exposures and joint target attainment in the Phase 3 patient population and to conduct PTA analyses in simulated patients with cIAI, cUTI, nosocomial pneumonia and VAP, using the joint PK/PD target described above [102]. Ceftazidime and avibactam steady-state PK exposure parameters and joint target attainment rates were compared across a range of clinical scenarios, including the presence/absence of systemic inflammatory response syndrome, bacteraemia or fever, white blood cell count (≤12,000/mm^3^ or >12,000/mm^3^) and various patient subgroups such as obesity, age, Acute Physiology and Chronic Health Evaluation II score and renal function categories (based on estimated creatinine clearance). With the exception of the 8–15 mL/min renal function group (which was limited by a small sample size of four patients), high joint target attainment rates (>93%) were attained for each indication in different clinical scenarios across patient subgroups [102].

PTA simulations using the final ceftazidime and avibactam population PK models were used to validate the approved ceftazidime–avibactam dosage regimen (2000 mg ceftazidime plus 500 mg avibactam 2-h IV infusions every 8 h), including adjustments for renal impairment. In these analyses, PTA values for target pathogens, including *P. aeruginosa* at MICs ≤8 mg/L, were 95–100% across indications and renal function groups; lower PTA values were associated with MICs of 16 and ≥32 mg/L [102]. These analyses also supported the current EUCAST and CLSI susceptible MIC breakpoints of ≤8 mg/L for ceftazidime–avibactam against *P. aeruginosa* [87,88,104]. Separate analyses evaluating the lung penetration of ceftazidime–avibactam have demonstrated linear PK in epithelial lining fluid in mice and humans, with approved doses achieving clinically-relevant exposures against diverse *P. aeruginosa* isolates in an infection murine model [107,108,109].

### 4.5. Ceftazidime–Avibactam in Clinical Trials

Two Phase 2 and five Phase 3, randomized, multicentre active-comparator trials have evaluated the efficacy and safety of ceftazidime–avibactam against carbapenems/best available therapy in adults hospitalized with serious Gram-negative infections (Table 3). Each trial, which enrolled patients with cIAI, cUTI or HAP including VAP, included a treatment period (5–21 days) and primary efficacy evaluations at a protocol-defined test-of-cure (TOC) visit [110,111,112,113,114,115,116]. Apart from REPRISE, which did not use formal statistical comparisons, non-inferiority of ceftazidime–avibactam versus the comparator treatment was demonstrated in the other four Phase III trials for their respective primary efficacy endpoints [112,113,114,115,116].

**Table 3:** Clinical cure and favourable microbiological response rates at TOC in Phase 2–3 clinical trials of ceftazidime–avibactam: patients with *P. aeruginosa* isolated at baseline.

	Clinical Cure, n/N (%)		Favourable Microbiological Response, n/N (%)	
	Ceftazidime– Avibactam	Comparator *	Ceftazidime– Avibactam	Comparator *
Phase 2 cIAI [110]				
ME population	NR	NR	5/5 (100)	5/5 (100)
Phase 2 cUTI [111]				
ME population	NR	NR	0/2 (0)	0/0
Phase 3 RECLAIM 1 and 2: cIAI [112]				
mMITT population	30/35 (85.7)	34/36 (94.4)	NR	NR
Phase 3 RECLAIM 3: cIAI [114]				
eME population	11/11 (100)	12/14 (85.7)	NR	NR
Phase 3 REPRISE: cIAI and cUTI [113]				
mMITT population: cIAI	1/1 (100)	1/1 (100)	NR	NR
mMITT population: cUTI	12/14 (86.0)	5/5 (100)	11/14 (79.0)	3/5 (60.0)
Phase 3 RECAPTURE 1 and 2: cUTI [115]				
mMITT population	NR	NR	12/18 (66.7)	15/20 (75.0)
Phase 3 REPROVE: HAP/VAP [116]				
mMITT population	22/39 (56.4)	19/26 (73.1)	22/58 (37.9)	18/47 (38.3)
ME population	16/24 (66.7)	14/18 (77.8)	13/31 (41.9)	12/28 (42.9)
eME population	NR	NR	18/42 (42.9)	14/35 (40.0)
CE population	27/42 (64.3)	27/35 (77.1)	NR	NR
Pooled Phase 3 (all indications)—MDR *P. aeruginosa* [117]				
mMITT population	NR	NR	32/56 (57.1)	21/39 (53.8)
Pooled Phase 3 (all indications)—*P. aeruginosa* bacteraemia [118]				
mMITT population	11/15 (73.3)	9/11 (81.8)	10/15 (66.7)	7/11 (63.6)

* Comparator agents were meropenem for Phase 2 cIAI; REPROVE and RECLAIM and imipenem–cilastatin for Phase 2 cUTI; doripenem for RECAPTURE; and the best available therapy for REPRISE. CE, clinically evaluable; cIAI, complicated intra-abdominal infection; cUTI, complicated urinary tract infection; eME, extended microbiologically evaluable; HAP, hospital-acquired pneumonia; ME, microbiologically evaluable; mMITT, microbiologically modified intention-to-treat population; NR, not reported; TOC, test-of-cure visit; VAP, ventilator-associated pneumonia. Clinical cure was defined as complete resolution or significant improvement of signs and symptoms of the index infection, with no further treatment required. Favourable microbiological response was defined as eradication/presumed eradication of original baseline pathogen(s).

Clinical and microbiological efficacy outcomes at TOC for the cohorts of patients with *P. aeruginosa* isolated at baseline in the Phase 2 and 3 adult trials are summarized in Table 3. Across the trials, ceftazidime–avibactam was generally effective in treating hospitalized adults with cUTI, cIAI and HAP/VAP caused by *P. aeruginosa*, as assessed by clinical cure and favourable microbiological response rates at the TOC visit [112,113,114,115,116]. In a pooled analysis of outcomes for patients with MDR Gram-negative isolates from the adult Phase 3 clinical trials, ceftazidime–avibactam demonstrated similar efficacy to comparators against MDR *P. aeruginosa* [117]. In the pooled microbiologically modified intention-to-treat (mMITT) population, a total of 56 patients in the ceftazidime–avibactam arm had MDR *P. aeruginosa* isolated at baseline. The ceftazidime–avibactam MIC range, MIC_50_ and MIC_90_ were 1 to >256, 8 and 64 mg/L, respectively, with 66.1% of isolates susceptible (MIC ≤ 8 mg/L). Favourable microbiological responses at TOC (pooled mMITT population) were observed in 32 of 56 (57.1%) patients in the ceftazidime–avibactam group and 21 of 39 patients (53.8%) in the comparator group [117]. For patients with bacteraemia due to *P. aeruginosa*, a pooled analysis of the five Phase 3 trials found that clinical and microbiological responses were similar to those in the overall set; among bacteraemic patients with *P. aeruginosa*, the response rates were somewhat lower, but similar between treatment groups [118]. Across the Phase 3 trials, 28-day mortality rates were between 0% and 9.6% (per-pathogen mortality rates have not been reported). Decreased susceptibility of some microbiological isolates to study treatments was reported in the REPROVE trial; however, for *P. aeruginosa,* 10 patients in the meropenem group and none in the ceftazidime–avibactam group had isolates with decreased susceptibility [116].

### 4.6. Real-World Experience

A growing body of literature reporting on real-world use of ceftazidime–avibactam infections is available and has recently been reviewed [119]; available publications (as of August 2021) reporting outcomes of *P. aeruginosa* infections treated with ceftazidime–avibactam are summarized in Table 4 [120,121,122,123,124,125,126,127,128,129,130,131]. Several other publications report aggregated outcomes for cohorts of patients with infections caused by other pathogens as well as *P. aeruginosa* or *Pseudomonas* species [132,133,134]. Most of these studies are limited by generally small samples and retrospective, non-comparative, observational designs. However, these data provide important insights into the real-world therapeutic effectiveness of ceftazidime–avibactam in the treatment of often severely ill patients with complicated and difficult-to-treat infections.

In the largest real-world study with specific data for *P. aeruginosa*, Jorgensen et al. (2019) evaluated 203 patients treated with ceftazidime–avibactam for ≥72 h at six US hospitals (2015–2019) for various MDR Gram-negative infections [122]. *P. aeruginosa* were isolated from 63 (31.0%) patients, and carbapenem-resistant *Enterobacterales* (CRE) from 117 (57.6%). The most common infection sources for *P. aeruginosa* were respiratory tract (60.3%), urinary tract (11.1%), osteoarticular (9.5%) and skin and soft tissue (9.5%). Among patients with *P. aeruginosa* infections, clinical failure, 30-day mortality and 30-day recurrence were reported in 19 (30.2%), 11 (17.5%) and 4 (6.3%) patients, respectively. 

Vena et al. (2020) reported on the outcomes of ceftazidime–avibactam treatment for 41 patients admitted to 13 Italian hospitals with infections caused by non-CRE MDR Gram-negative bacteria, most commonly HAP (48.8%), primary bacteraemia (17.1%), IAI (9.8%) and bone infections (9.8%) [130]. Thirty-three patients (80.5%) had monomicrobial *P. aeruginosa* infections and four patients (9.8%) had polymicrobial infections with *P. aeruginosa* and ESBL-positive *Enterobacterales* or *Acinetobacter baumannii*. Patients started ceftazidime–avibactam therapy subsequent to development of antimicrobial resistance to prior antibiotic therapy (61.0%) or failure of prior antibiotic therapy (34.1%) and median treatment duration was 13 days. Most patients (80.5%) received ceftazidime–avibactam as combination therapy. Clinical cure was achieved in 29/33 (87.9%) and 4/4 (100%) of patients with monomicrobial and polymicrobial *P. aeruginosa* infections, respectively, and in 4/4 (100%) of patients with monomicrobial ESBL-producing *Enterobacterales* infections. Development of resistance to ceftazidime–avibactam was not detected in any of 61 patients with repeat testing data available.

In another example of the efficacy of ceftazidime–avibactam in polymicrobial *P. aeruginosa* and *Enterobacterales* infections, Gofman et al. (2018) reported on a patient with polymicrobial ventriculitis caused by *P. aeruginosa* and carbapenem-resistant *K. pneumoniae*, who was successfully treated with ceftazidime–avibactam and intrathecal amikacin [121].

Santevecchi et al. (2018) evaluated 10 patients treated with ceftazidime–avibactam at a US hospital for non-*K. pneumoniae* infections during 2015–2016 [128]. Primary infections included pneumonia (6/13; 46%), skin/soft tissue (3/13; 23%), bacteraemia (2/13; 15%) and IAI (2/13; 15%); three patients were classified as having more than one infection. *P. aeruginosa* was the most commonly isolated organism (8 of 21 isolates). Five patients (50%) received ceftazidime–avibactam monotherapy. Microbiological cure was achieved in 6/9 evaluable patients (67%) and clinical success in 7/10 patients (70%). Resistance emergence to ceftazidime–avibactam was reported in 2/10 patients (20%), one of whom was infected with *P. aeruginosa.*

Rodriguez-Nunez et al. (2018) evaluated outcomes for eight patients with infections caused by MDR or XDR *P. aeruginosa* admitted to a teaching hospital in Spain (2016–2017) treated with ceftazidime–avibactam for ≥72 h [127]. Infection sources were HAP in four patients (50.0%) and tracheobronchitis, osteomyelitis, meningitis and catheter-related bacteraemia in one patient each. The clinical cure rate was 50%; 30-day and 90-day mortality rates were 13% and 38%, respectively; no cases of ceftazidime–avibactam resistance emergence were reported.

Metafuni et al. (2019) and Xipell et al. (2017) have reported positive outcomes in individual patients with severe drug-resistant *P. aeruginosa* infections treated with ceftazidime–avibactam without documented emergence of resistance [126,131].

Spoletini et al. (2019) reported on eight adults with CF who received a total of 15 courses of ceftazidime–avibactam for pulmonary exacerbations not responding to conventional antibiotic treatment [129]. Four patients were colonized with *P. aeruginosa*, two with *Burkholderia cepacia* complex and two with both pathogens; and five were on the active waiting list for lung transplantation. Treatment with ceftazidime–avibactam was associated with an effective clinical response in 13 of 15 (86.7%) treatment courses. Four of six patients with *P. aeruginosa* infections who had been suspended from the active transplant list were reactivated following clinical stabilization; one patient received a successful transplant while on treatment, and one who was on the transplant list died whilst on ceftazidime–avibactam due to respiratory failure. No cases of ceftazidime–avibactam resistance emergence were reported.

**Table 4:** Real-world experience with ceftazidime–avibactam in patients with *P. aeruginosa* infections.

Study	Patient Characteristics	Baseline Pathogens (Resistance Mechanisms)	Ceftazidime– Avibactam Dose and Duration	Concomitant Antibiotics, n/N (%)	Reported Outcomes
Algwizani (2018) [120]	6 male patients, age 15–87 years 2/6 (33%) patients had bacteraemia	CRPA (*n* = 3) CRKP (*n* = 3; 2 with OXA-48, 1 with NDM and OXA-48)	2.5 g q8h, adjusted for renal function Range 9–30 days	3/6 (50%)	5/6 (83%) patients achieved clinical and/or microbiological cure, including 3/3 (100%) with CRPA infections. 1 patient (17%) died 9 days after starting ceftazidime–avibactam treatment (NDM and OXA-48 *K. pneumoniae* CLABSI and VAP).
Gofman (2018) [121]	32-year-old male with intracranial haemorrhage due to traumatic injury; ventriculitis and sepsis	*P. aeruginosa* CRKP *Streptococcus* *viridans*	2.5 g q8h, 6 weeks	1/1 (100%)	CSF cultures were sterile after 3 days’ treatment with ceftazidime–avibactam + intrathecal amikacin, with treatment continued for 4 and 6 weeks, respectively. The patient did not experience any seizures or neurological deficits and was transferred to a long-term care facility for rehabilitation.
Jorgensen (2019) [122]	203 patients who received ceftazidime–avibactam for >72 h, median age 62 years, 62% male 22/203 (11%) patients had bacteraemia	CRE (58%); *Pseudomonas* spp. (31%); others (23%)	92/203 patients (45%) required renal dose adjustments; median duration 9 days	68/203 (34%) overall 20/63 (30%) in patients with *Pseudomonas* spp. infection	Clinical failure occurred in 59/203 (29%) patients overall and 19/63 (30%) in patients with *Pseudomonas* spp. Infection. 30-day recurrence occurred in 12/203 (6%) patients overall and 19/63 (6%) in patients with *Pseudomonas* spp. Infection.
King (2016) [123]	10 patients, mean age 73 years, 70% male, median CCI 6 1/10 (10%) patients had bacteraemia	*P. aeruginosa* (MDR and XDR)	NR	5/10 (50%)	Microbiological cure achieved in 9/10 (90%) patients. Clinical success achieved in 8/10 (80%) patients.
Kuang (2020) [124]	20 patients, mean age 55 years, 70% male, mean CCI 4 7/20 (35%) patients had bacteraemia	*K. pneumoniae* (*n* = 18; 12 CRKP) *P. aeruginosa* (*n* = 3; 2 car-bapenem-resistant) *Escherichia coli* (*n* = 3; all ESBL-producing strains) Others (*n* = 9)	Standard dose, adjusted for renal function, duration NR	10/20 (50%)	Clinical cure and failure at 30 days were achieved in 9 and 11 cases, respectively, including 2/3 (66%) of the patients with *P. aeruginosa* infection and 1/3 (33%) of the *P. aeruginosa* patients with HAP and cIAI and septic shock; co-infected with CRKP and *E. coli* (ESBL); and treated with ceftazidime–avibactam, tigecycline and aztreonam died after 3 days of treatment. Adverse effects reported in 3/20 (15%) of patients.
Meschiari (2020) [125]	3 patients (age 29–66 years, 2 male) with neurosurgical infections Patients 1 and 3 (both male) were treated with ceftazidime–avibactam (patient 1 after switching from ceftolozane–tazobactam)	XDR *P. aeruginosa* (*n* = 3) KPC-KP (*n* = 1)	Patient 1: 2.5 g q6h extended infusion (off-label dose) Patient 3: 2.5 g q8h	Patient 1: az-treonam 2 g q6h for 6 weeks Patient 3: col-istin then switch to az-treonam 2 g q6h for 8 weeks	Both patients treated with ceftazidime–avibactam achieved complete resolution of vertebral osteomyelitis by CT/MRI after 60 days. Rectal swab performed for routine screening at the end of treatment (Patient 3) yielded XDR *P. aeruginosa* with acquired resistance to ceftazidime–avibactam (MIC = 16 mg/L).
Metafuni (2019) [126]	3 haematological patients with neu-tropenia and Gram-negative bac-teraemia, ages 52–69 years, all male, CCI = 3	KPC-KP (*n* = 2) MDR *P. aeruginosa* (*n* = 1)	2.5 g q8h, add-ed to current antibiotics combination Median (range) 15 (12–16) days	3/3 (100%)	Clinical success: 2/3 (67%) of patients, including 1/1 (100%) patient with *P. aeruginosa* in-fection.
Rodríguez-Núñez (2018) [127]	8 patients, ages 51–71 years, 88% male 1/8 (13%) patients had bacteraemia	*P. aeruginosa* (MDR and XDR)	Dose NR, range 7–34 days	6/8 (75%)	Clinical cure achieved in 4/8 (50%) of patients. 30-day mortality: 1/8 (13%). 90-day mortality: 3/8 (38%). 1/8 (13%) of patients devel-oped encephalopathy that im-proved with drug discontinua-tion.
Santevecchi (2018) [128]	10 patients, ages 32–74 years, 50% male 8 patients had renal impairment, in-cluding 4 undergo-ing CRRT	MDR *P. aeruginosa* (*n* = 8) CRE (*n* = 9) Other (*n* = 4)	Doses NR, ad-justed for renal function Median (range) 16 (4–50) days	5/10 (50%)	Clinical success: 7/10 (70%) patients, including 7/8 (88%) patients with *P. aeruginosa* in-fection. Microbiological cure: 6/9 (67%) patients, including 6/8 (75%) patients with *P. aeruginosa* in-fection. 2/10 patients (20%) developed emergence of resistance while on therapy with ceftazidime–avibactam.
Spoletini (2019) [129]	8 patients with CF (63% female, ages 22–41 years) re-ceived 15 courses of ceftazidime–avibactam (1–4 courses/patient)	MDR *P. aeruginosa* (*n* = 6) Other (*n* = 4)	Doses NR Range 12–145 days	8/8 (100%)	Effective clinical response seen in 13/15 courses (87%), in-cluding 10/11 where *P. aeruginosa* was identified in spu-tum. 2/8 (25%) of patients with a very poor prognosis died ow-ing to complex underlying lung pathology.

CCI, Charlson Comorbidity Index score; CRE, carbapenem-resistant *Enterobacterales*; CRKP, carbapenem-resistant *Klebsiella pneumoniae*; CRRT, continuous renal replacement therapy; CT, computed tomography; KPC-KP, *K. pneumoniae* carbapenemase-producing *K. pneumoniae*; MDR, multidrug-resistant; MIC, minimum inhibitory concentration; NR, not reported; q8h, every 8 h; UTI, urinary tract infection; XDR, extensively drug-resistant.

## 5. Conclusions

*P. aeruginosa* infections can be challenging to treat, as the species has limited intrinsic susceptibility to many antibiotics as well as great propensity to express further multiple resistance mechanisms through mutation and horizonal gene acquisition [41,42]. *P. aeruginosa* is relatively common in infections in healthcare settings, causing around 10–20% of skin, lower respiratory and urinary tract infections in hospitalized patients, and is particularly associated with severe and critical illness, such as in ICU and haematological patients. Patients with acute *P. aeruginosa* infections are at significantly greater risk of 30-day mortality when receiving inappropriate initial antimicrobial therapy (IAT) vs. those receiving appropriate IAT; however, selection of appropriate IAT in some settings and regions is challenged by increasing antimicrobial resistance, including MDR and DTR *P. aeruginosa* [135,136].

Ceftazidime–avibactam demonstrates good in vitro activity against *P. aeruginosa* relative to comparator β-lactam agents, aminoglycosides and fluoroquinolones [79,89,90,91,92,97], with susceptibility rates comparable to amikacin and ceftolozane–tazobactam [79,90,91,94,97]. Ceftazidime–avibactam is not active against MBL-producing pathogens, and *P. aeruginosa* is capable of expressing multiple resistance mechanisms (including MBLs) that render some isolates, particularly MDR strains, non-susceptible to ceftazidime–avibactam. Accordingly, as with all antimicrobials, ceftazidime–avibactam usage should be guided by local susceptibility patterns and microbiological/antibiogram data whenever possible.

In Phase 3 clinical trials, ceftazidime–avibactam was associated with generally similar clinical and microbiological outcomes to comparators (carbapenems/best available therapy) in adult patients with cIAI, cUTI or HAP/VAP caused by *P. aeruginosa*, including ceftazidime non-susceptible and MDR strains [112,113,114,115,116,117]. In population PK modelling and exposure simulations of patients with cIAI, cUTI or HAP/VAP, >95% PTA was predicted for approved ceftazidime–avibactam dosage regimens (2000 mg ceftazidime plus 500 mg avibactam 2-h IV infusions every 8 h, adjusted for renal function) against *P. aeruginosa* with MICs ≤ 8 mg/L [104]. While there are relatively few published real-world data for ceftazidime–avibactam treatment of serious *P. aeruginosa* infections, favourable outcomes with ceftazidime–avibactam treatment have been reported in some patients with infections caused by MDR and XDR *P. aeruginosa*, without documented resistance emergence (albeit from a small sample of anecdotal reports) [121,122,126,127,128,129,130,131]. The efficacy and safety of ceftazidime–avibactam in patients with CF have not been evaluated in randomized controlled trials; however, available in vitro and real-world data suggest a potential role for this agent in managing acute *P. aeruginosa* infections in cases where other antibiotics have failed [92,129], subject to local/institutional formularies and national product labelling. 

As the risk factors for infections with different MDR bacteria, including *P. aeruginosa* and *Enterobacterales*, are often the same, ceftazidime–avibactam offers a good empiric treatment option for patients considered at risk of MDR Gram-negative infections, including those caused by non MBL-producing MDR and DTR *P. aeruginosa*, and its potential role in such settings is recognized in various national and international treatment guidelines [59,62,63,64]. Appropriate use of all antibiotics, including ceftazidime–avibactam, guided by diagnostic susceptibility data (where available) and knowledge of local resistance patterns, are vital to support antimicrobial stewardship and limit the emergence and spread of resistance.

## Data Availability

No new data were created or analysed in this review article. Data sharing is not applicable to this article.
